# Affinity-purified DNA-based mutation profiles of endometriosis-related ovarian neoplasms in Japanese patients

**DOI:** 10.18632/oncotarget.24546

**Published:** 2018-02-22

**Authors:** Masako Ishikawa, Kentaro Nakayama, Kohei Nakamura, Ruriko Ono, Kaori Sanuki, Hitomi Yamashita, Tomoka Ishibashi, Toshiko Minamoto, Kouji Iida, Sultana Razia, Noriyoshi Ishikawa, Satoru Kyo

**Affiliations:** ^1^ Department of Obstetrics and Gynecology, Shimane University School of Medicine, 6938501 Izumo, Japan; ^2^ Department of Organ Pathology, Shimane University School of Medicine, 6938501 Izumo, Japan

**Keywords:** ovarian clear cell carcinoma, ovarian endometrioid carcinoma, *ARID1A*, *POLE*, liquid microdissection

## Abstract

**Aim:**

Endometriosis-related ovarian neoplasms (ERONs) have recently attracted considerable attention; however, the prevalence and patterns of *ARID1A* and *POLE* mutations in ERONs have not been studied in detail. The aim of this study was to investigate not only the carcinogenesis of ERONs, but also the prognostic significance of several gene mutations in this cohort. We used DNA purified from only tumor epithelial cells, from which fibroblasts were removed, using a specific method we called “liquid microdissection”.

**Methods:**

Tissue samples from 22 ovarian carcinomas (13 endometrioid, and nine clear cell) were used. Tumor cells were isolated using a cell sorting system and DNA was purified from tumor epithelial cells. Nucleotide sequencing was conducted to analyze the mutational status of *ARID1A, p53, PTEN, POLE, PIK3CA*, and *KRAS*.

**Results:**

In ERONs, the frequencies of somatic mutations in *ARID1A, p53, POLE, PTEN, PIK3CA*, and *KRAS* were 19/20 (95.0%), 7/19 (36.8%), 9/22 (40.9%), 13/19 (68.4%), 3/19 (15.8%), and 1/9 (11.1%). The frequency of *ARID1A* mutations was significantly higher than that reported previously. Kaplan-Meier survival analysis revealed that mutations in all genes, including *POLE*, were not associated with patient prognosis in our Japanese cohort.

**Conclusions:**

Our results suggest that the frequency of *ARID1A* mutations in ERONs may be higher than that previously reported. In addition, the “liquid microdissection” method that we chose for DNA purification could be used to obtain high-quality sequencing results. The findings suggest that *ARID1A* mutations represent the basis of ERON carcinogenesis; other subsequent gene mutations may result in the progression of carcinogenesis.

## INTRODUCTION

Ovarian cancer (OC) is the most lethal gynecologic malignancy [1]. In Japan, ovarian clear cell carcinoma (OCCC) is the second most common histologic subtype of ovarian cancer [2]. S everal reports have described an increase prevalence of OCCC in Japan compared with that in other countries [2, 3]; however, the reason for this is unknown. Previously, various studies have demonstrated an association between OCCC or ovarian endometrioid carcinoma (OEC) and endometriosis, and it is widely recognized that gene alterations associated with carcinogenesis occur in endometriosis. Common molecular genetic alterations in endometriosis-related ovarian neoplasms (ERONs) have already described [4–12], [15–18].

Recently, several reports have suggested that numerous genetic alterations are associated with carcinogenesis, leading from endometriosis to ovarian cancer. A *KRAS* mutation has been detected in OEC tissue, but not in atypical endometriosis bordering the cancerous region [8]. Common molecular genetic alterations in ERON, such as *PTEN* deletion and microsatellite instability, may also be detected in normal-appearing epithelial cells of endometriotic cysts [7, 9]. Among all molecular genetic changes identified to date, inactivating mutations of the *ARID1A* tumor suppressor gene are the most common in ERON. The role of *ARID1A* alterations in the early molecular pathogenesis of OCCC is demonstrated [10, 11]. Furthermore, it was identified that atypical endometriosis and OCCC share molecular alterations, such as inactivating mutations for *ARID1A*, activating mutations for *PIK3CA*, and the hypomethylation of HNF1 homeobox B (*HNF1B*) [12].

Thus, molecular biological analyses of ERONs have identified several molecular genetic alterations in *ARID1A*, *PTEN*, *PIK3CA*, *KRAS*, as well as in other genes. Additionally, numerous reports describe the relationship of carcinogenesis from endometriosis to OEC or OCCC; however, much less is known about the pathogenetic pathways and the order in which alterations occur. Moreover, the correct frequency of gene alterations could not be determined as the DNA used in these studies was purified by several different methods.

Recently, a novel treatment strategy, in which inhibition of enhancer of zeste homology 2 (*EZH2*) activity electively suppressed the growth of *ARID1A-*mutated OCCC cells, has been reported [13]. *EZH2* is the functional enzymatic component of the Polycomb Repressive Complex 2 (PRC2), which is responsible for healthy embryonic development through the epigenetic maintenance of genes responsible for regulating development and differentiation [14].

GSK126, a highly specific EZH2 inhibitor, caused the regression of established *ARID1A*-mutated OCCC and decreased the number of disseminated tumor nodules in xenograft models [13]. Thus, GSK126exhibits potential as a molecular targeted drug that inhibits the proliferation of *ARID1A-*mutated ovarian clear cell carcinoma cells by targeting and inhibiting *EZH2*. The clinical application of drugs that target and inhibit *EZH2* require further examination of the status of *ARID1A* mutations in OCCC. *ARID1A* is a large gene that contains 20 exons, and mutations are distributed evenly across the whole gene [15, 16]. It has already been shown in previous studies that the *ARID1A* mutation frequency in patients with OCCC and OEC hadis about 30-60% [15, 16]. For the utilization of EZH2 to the advantage of patients, the correct frequency of *ARID1A* mutation must be considered. Here, we attempted to show that the frequency of the *ARID1A* mutation is higher than that previously reported, using pure DNA purified from tumor epithelial cells, using a method we termed ‘liquid microdissection’.

Polymerase epsilon (*POLE*), which has recently become the focus for endometrial carcinoma, is a DNA polymerase with a proofreading exonuclease domain. It is responsible for the recognition and excision of mispaired bases, thereby allowing high-fidelity DNA replication. The Cancer Genome Atlas (TCGA) research network recently identified an ultra-mutated group of endometrial carcinomas characterized by mutations in *POLE* and exceptionally high substitution rates [17]. Previous reports have demonstrated the prevalence and patterns of *POLE* mutations in OEC [18]; however, these have not been studied in detail in other histological types. In comparison, relatively little known is known about the *POLE* mutation status of ERONs.

However, molecular analyses remain challenging: it is difficult to obtain DNA from pure tumor cells and to avoid contamination from fibroblasts. As such, no reports have utilized purified tumor samples to determine sequence mutations in ERONs. The analysis of such genetic alterations is typically complicated by contamination of fibroblast DNA. Thus, the quality of DNA products and the sensitivity of detection techniques for analyzing ovarian carcinomas must be improved. In this study, we purified DNA from only tumor epithelial cells after removing fibroblasts from seeded primary cultured cells [19–25]. We called this method “liquid microdissection.” Using this technique, we investigated the carcinogenesis of OEC and OCCC using high-quality purified DNA.

## RESULTS

### Clinical and pathological features

The clinical and histological features of nine clear cell and 13 endometrioid ovarian carcinomas are described (Table 1). Patient age ranged from 47 to 80 years of age with an average of 58.0 years, and all patients were post-menopausal. Fourteen patients had FIGO (2009) stage 1 disease, one patient had stage 2 disease, four patients had stage 3 disease, and three had stage 4 disease.

**Table 1:** Clinical characteristics of patients with ERONs

Case no.	Histology	Age (years)	FIGO Stage	Residual tumor	Recurrence		*ARID1A*	*p53*	*POLE*	*PTEN*	*PIK3CA*	*KRAS*
1	E	58	IIIC	N	Y		M	WT	M	WT	WT	NA
2	E	57	IIIC	Y	Y		M	WT	WT	WT	WT	NA
3	E	72	IIIC	N	Y		M	M	M	M	WT	WT
4	E	60	IV	Y	Y		M	M	M	NA	WT	WT
5	E	80	IA	N	Y		WT	WT	WT	NA	WT	WT
6	E	59	IC	N	N		M	WT	WT	M	M	WT
7	E	58	IA	N	N		M	WT	WT	M	M	WT
8	E	61	IIC	N	N		M	WT	M	M	WT	NA
9	E	50	IC	N	N		M	M	WT	M	WT	M
10	E	47	IC	N	N		M	WT	M	WT	WT	NA
11	E	77	IC	N	N		M	M	M	M	WT	W
12	E	58	IC	N	N		M	WT	WT	M	WT	NA
13	E	76	IC	N	N		M	M	WT	NA	WT	NA
14	C	56	IV	Y	Y		M	NA	M	WT	NA	NA
15	C	63	IC	N	Y		M	WT	WT	M	WT	NA
16	C	55	IIIC	Y	Y		M	M	M	M	M	WT
17	C	50	IC	N	N		NA	NA	WT	WT	NA	NA
18	C	62	IC	N	Y		M	WT	M	M	WT	NA
19	C	61	IV	Y	Y		M	M	WT	M	WT	WT
20	C	50	IC	N	N		NA	NA	WT	WT	NA	NA
21	C	71	IC	N	N		M	WT	WT	M	WT	WT
22	C	55	IC	N	Y		M	WT	WT	M	WT	NA
					Mutation frequency %		95	36.8	40.9	68.4	15.8	11.1
					Previous Reports %	E C	30^(15) (16)^ 46-57^(15)(16)^	9^(20)(21)^ 25^(20)(21)^	20^(18)^ 8^(18)^	8^(7)(9)^	20^(22) (23)^ 33-46^(22 )(23)^	10^(24)(25)^

NA: Not available.

WT: Wild type.

M: Mutation.

E: endometroid carcinoma.

C: clear cell carcinoma.

### Identification of *ARID1A, p53, POLE, PTEN, PIK3CA*, and *KRAS* mutations

The mutational status of *ARID1A, p53, POLE, PTEN, PIK3CA*, and *KRAS* in all 22 purified ovarian tumors is summarized in Table 1. The frequency of mutations of these genes were described comparing previous reports [7] [9] [15, 16] [18] [20–25]. Somatic mutations in *ARID1A* were identified in 19 (95.0 %) of 20 ERONs. Somatic mutations in *POLE* were identified in nine (40.9 %) of 22 ERONs. The mutations in *ARID1A* and *POLE*, and the fact that they were somatic, were confirmed by Sanger sequencing of DNA of tumor and normal tissues from the corresponding patients (Supplementary Figure 1). The frequency of *ARID1A* mutations was higher than that in previous reports, especially for clear cell carcinoma. These mutational types are described in Supplementary Table 2.

### Clinical features of ERONs with POLE mutations

Univariate analysis of clinicopathologic factors showed that *POLE* mutations were only related to FIGO stage (Table 2). We then investigated the statistical correlation between mutations in these genes and *POLE* mutations (Table 3). Among these genes, there were no significant correlations regarding the mutation status.

**Table 2:** Association between *POL*E mutation and clinicopathological factors in patients with ERONs

Factors	Patiens	*POLE* mutation		*P*
		Negative	Positive	
FIGO stage				
I, II	13	11	4	0.047
III, IV	9	2	5	
Age (y)				
<60	12	8	4	0.429
≧60	10	5	5	
Residual tumor				
Negative	17	11	6	0.323
Positive	5	2	3	
Recurrence				
Yes	11	5	6	0.193
No	11	8	3	
Secondline treatment sensitivity				
Yes	3	1	3	0.303
No	8	4	3	
Dead of disease				
Yes	8	5	3	0.806
No	14	8	6	

**Table 3:** Association between *POLE* mutation and other gene mutations in patients with ERONs

Factors	Patiens	*POLE* mutation		*P*
		Negative	Positive	
*ARID1A*				
Negative	1	1	0	0.353
positive	19	10	9	
*p53*				
Negative	12	8	4	0.311
positive	7	3	4	
*PTEN*				
Negative	6	3	3	0.636
positive	13	8	5	
*PIK3CA*				
Negative	16	9	7	0.737
positive	3	2	1	
*KRAS*				
Negative	9	5	4	0.389
positive	1	1	0	

**Figure 1:**
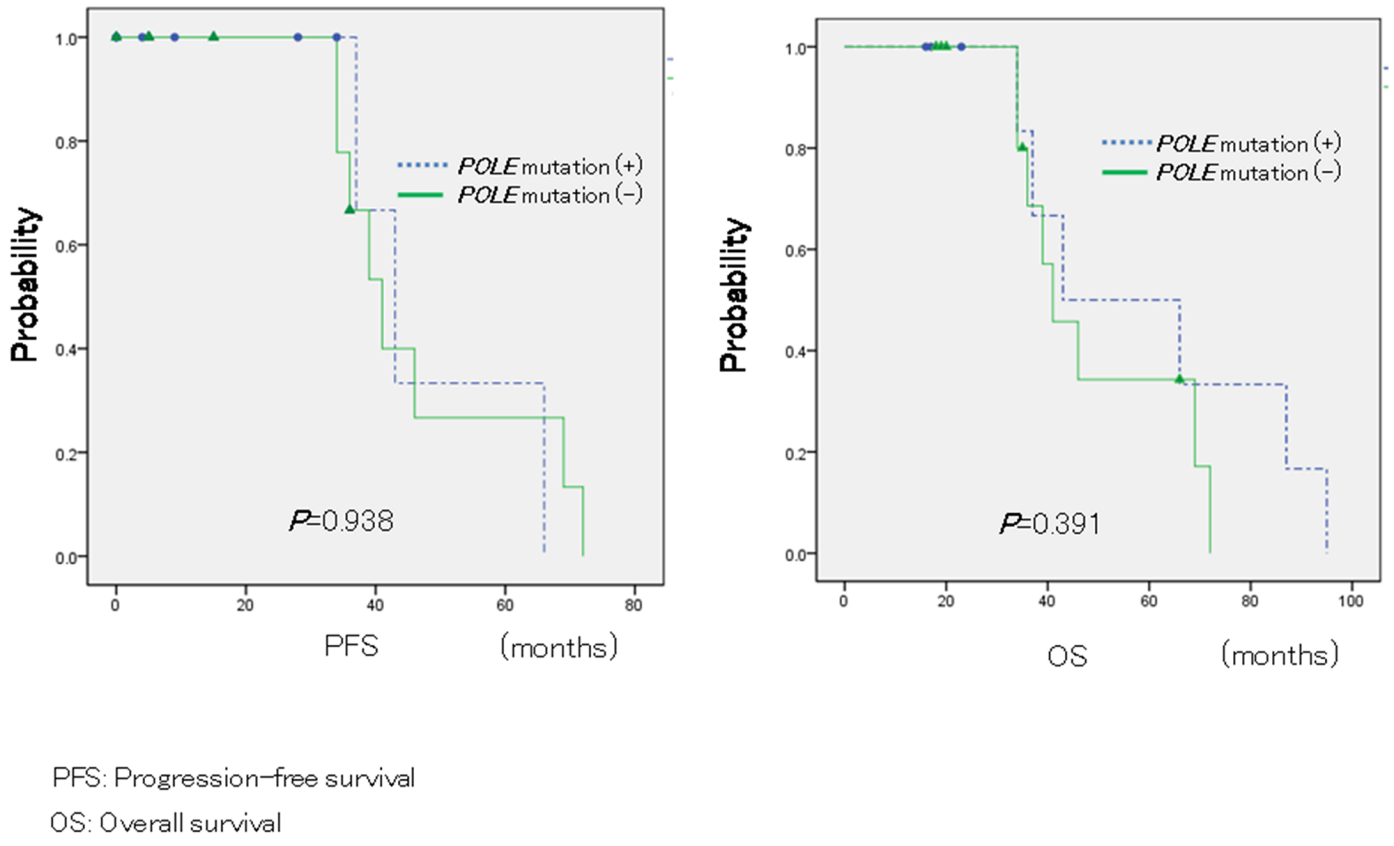
Relationship between *POLE* mutations and patient prognosis in ovarian carcinomas In 22 patients with endometrioid and clear cell carcinoma, Kaplan-Meier survival analysis was performed, showing that *POLE* mutation positivity (blue line; n = 9) and negativity (green line; n = 11) are not associated with progression-free survival and overall survival (*P* = 0.938, log-rank test; *P* = 0.391, log-rank test).

Finally, Kaplan-Meier analyses were performed to determine potential correlations between *POLE* mutations and patient prognosis. However, Kaplan-Meier analysis similarly did not show a significant difference for PFS and OS (*P* = 0.938; log-rank test; *P* = 0.391, log-rank test; Figure 1).

## DISCUSSION

The present study delivers three major findings. First, we showed that the prevalence of *ARID1A* mutations in ERONs is 95.0% (19/20). Previous reports showed rates of 30% in OEC and 46–57% in OCCC [15, 16]. At the onset of this study, we were concerned about complications due to the contamination of fibroblasts that were present with tumor epithelial cells in primary cultures from tumor tissue. In studies that examine gene alterations in various tumors, it is important to ensure robust DNA quality. In this study, DNA was purified from only tumor epithelial cells, which were isolated using a cell sorting system. Our results showed that DNA purified by “liquid microdissection” from tumor tissue could be used to identify the exact prevalence of *ARID1A* mutations, which was found to be higher than that stated in previous reports. We used a small sample in this study; however, it should be emphasized that this limitation was overcome by using a strategy for the purification of high-quality DNA from tissue culture sorting without contamination of fibroblasts, which makes the present findings more robust.

ERONs are the most important ovarian tumors in Japanese patients, based on the enhanced prevalence in this population [7, 26]. In Japan, a study showed that endometriosis-related ovarian carcinoma (ERON) is not rare, with an estimated incidence of 0.72% [27]. The carcinogenesis of ERONs from the endometriosis stage onwards was unclear; however, recently, several reports have demonstrated the carcinogenesis of ERONs. In a model of genetically engineered mice harboring an oncogenic allele of *KRAS* resulting in benign lesions reminiscent of endometriosis, a deletion of *PTEN* caused progression towards the OEC, but not the OCCC [28]. The role of *ARID1A* alterations in early molecular pathogenesis of CCC has been demonstrated in two recent reports [10, 11].

Regarding the carcinogenesis of ERONs, it is not clear which step is essential for the initiation or progression from benign tumor to cancer. The actual frequencies of certain genetic alterations have also not beendetermined. As *ARID1A* mutations are detected in endometrioma, it has been suggested that they are involved in the onset and progression of cancer [29]. Thus, based on the observed high frequency of *ARID1A* mutations, we suggest that such alterations form the basis of carcinogenesis for ERONs, and that other gene mutations might be gained subsequently to facilitate the progression of carcinogenesis.

The identification of every association between carcinogenesis and gene alteration of ERONs was impossible in the present study alone. There are some limitations to this study: it is difficult to generalize whether a high mutation frequency leads to carcinogenesis. In this study, a large number of mutations were detected in *ARID1A.* With regard to this, there are various types of mutations, which may be either pathogenic or of uncertain importance. For example, variants of uncertain significance (VUS) were reported among*BRCA1/2* gene alterations upon examining a HBOC family cohort [30]. The identification of the specific alteration that leads to pathogenic change requires further investigation. The identification of pathogenic VUS requires the accumulation of large amounts of VUS data.

Another limitation is that there are numerous gene alterations that result not only from gene mutations, but are also affected by micro RNA, gene methylation, and so on. In this study, we only performed mutational analysis by Sanger sequencing, which may be insufficient to identify epigenetic changesassociated with carcinogenesis. Further studies will be needed to be established the association between carcinogenesis and epigenetic changes in this context.

Second, we evaluated the clinicopathological and prognostic significance of *POLE* mutations in ERONs. *POLE* is a DNA polymerase with a proofreading exonuclease domain, and is responsible for the detection and excision of mispaired bases, thus facilitating high-accuracy DNA replication [31]. In the TCGA series, *POLE* mutations were found in 3% of colorectal cancers [32] and 7% of endometrial cancers (ECs). Improved PFS in patients with *POLE* ultra-mutated EC has been reported [17]. Recently, Hoang et al [18] described that *POLE* mutations are found in 6% of low-grade and 17% of high-grade endometrioid carcinomas in OC. However, little is known about the *POLE* mutation status in ERONs.

In the present study, there was no significant relationship between *POLE* mutations and PFS or OS. We first reported the relationship between *POLE* mutations and patient prognosis in ERONs in the current study. Prior to this study, we hypothesized that patients with *POLE* mutations how good prognosis. In this study, some patients with *POLE* mutations exhibited good response to second-line chemotherapy with disease recurrence (date not shown). However, because of the small sample size, we did not have sufficient statistical power to determine its prognostic significance for ERONs. In the future, studies with a greater number of cases will be needed to determine the prognostic significance of *POLE* mutations.

There was another limitation regarding the mutation analysis of *POLE*. We could not confirm which mutations are responsible for the pathogenesis of the disease. In addition, hot spot mutations represent a possible reason for the difference in results between our study and TCGA data. Zou et al [33] recently reported the same missense mutations in *POLE* at p.S297F in OEC in a Chinese population. They were also unable to identify any hot spot mutations that are frequently identified in endometrioid EC based on TCGA data [17]. Mutation hot spots might be different between races or types of cancer. The results are also difficult to interpret based on the fact that mutations other than hot spot mutations have not been described in detail. Whether the mutation is significant could be defined by functional or configuration analyses. If the role of several mutations could be precisely proven, it may be possible to better infer correlations between *POLE* mutations and patient prognosis.

Recently, Church et al [34, 35] reported *POLE*-ultra-mutated and MSI groups serve as biomarkers of the blockade of immune checkpoints in cancer immunotherapy. These two groups are characterized by an active immune microenvironment. The interaction between programmed death 1 (PD-1) receptor and programmed death ligand 1 (PD-L1) is an important pathway for inhibiting the immune checkpoint system. These pathways are already accepted as a target for melanoma and lung cancer [36, 37]. Hamanishi et al [38] also reported that the anti-PD-1 antibody nivolumab has positive effects on recurrent ovarian cancer, especially for clear cell carcinoma. Therein, a patient with multiple peritoneal dissemination exhibited complete response without recurrence after treatment with nivolumab. We expect that an anti-PD-1 antibody might show efficacy in patients with ovarian carcinoma such as clear cell carcinoma from ERONs. The mechanism associated with the effect of the anti-PD-1 antibody was also previously demonstrated [38].

Previous studies noted that *POLE*-mutated ECs are typically associated with strong lymphocyte infiltration [34, 35, 39]; thus, patients with *POLE*-ultra mutated and MSI ECs may receive the maximum benefit from drugs such as immuno-checkpoint inhibitors [40]. Overexpression of PD-1/PD-L1 or loss of MMR proteins might be associated with response to checkpoint blockade immunotherapies; thus, these factors potentially represent useful biomarkers.

After the initial TCGA report, multiple studies have demonstrated that *POLE*-ultra mutated ECs are characterized by excellent prognosis, despite the high histological grade; however, the reasons for such favorable outcomes are not completely understood [35, 39, 41, 42]. In the future, research on biomarkers that predict response to checkpoint blockade immunotherapies could provide the main benefit in terms of durable responses or survival. If *POLE*-mutated ERONs also have excellent prognosis, this knowledge will likely affect the course of immunotherapy for these patients, and perhaps lead to investigation into treatment using immuno-checkpoint inhibitors for ERONs with disease recurrence. Overexpression of PD-1/PD-L1 or loss of MMR proteins might be associated with the response to checkpoint blockade immunotherapies; thus, these markers would be useful biomarkers. We aim to conduct a further examination of the association between checkpoint blockade immunotherapies and *POLE* mutations.

Finally, in the present study, somatic mutations in both *ARID1A* and *p53* were identified in four (40.0%) of 10 endometrioid carcinomas and in two (33.3%) of six clear cell carcinomas. A significant correlation between both *ARID1A* and *p53* mutations and PFS was described for OCCC (*p* = 0.025, log-rank test); however, this was not described for OEC (*p* = 0.687, log-rank test) (data not shown). In previous reports, *ARID1A* was shown to be mutually exclusive of *p53* [43, 44]; however, in recent reports, *ARID1A* mutations and *p53* mutations have been described in the same case [45]. The explanation for this has been unclear; however, their gene products have been shown to form a complex that regulates the transcription of CDKN1A and SMAD4 [43]. This suggests that mutations in these genes may be sufficient to promote carcinogenesis through a common pathway.

In summary, the frequency of *ARID1A* mutations in ERONs may be higher than that reported previously. The “liquid microdissection” method, which was performed for the purification of DNA, yields high-quality sequencing results. *ARIDA* mutations may be the basis for carcinogenesis in ERONs; we speculate that other gene mutations are gained subsequently, thus facilitating the progression of carcinogenesis.

## MATERIALS AND METHODS

### Tissue samples and tumor cell isolation: cell sorting

**Figure 2:**
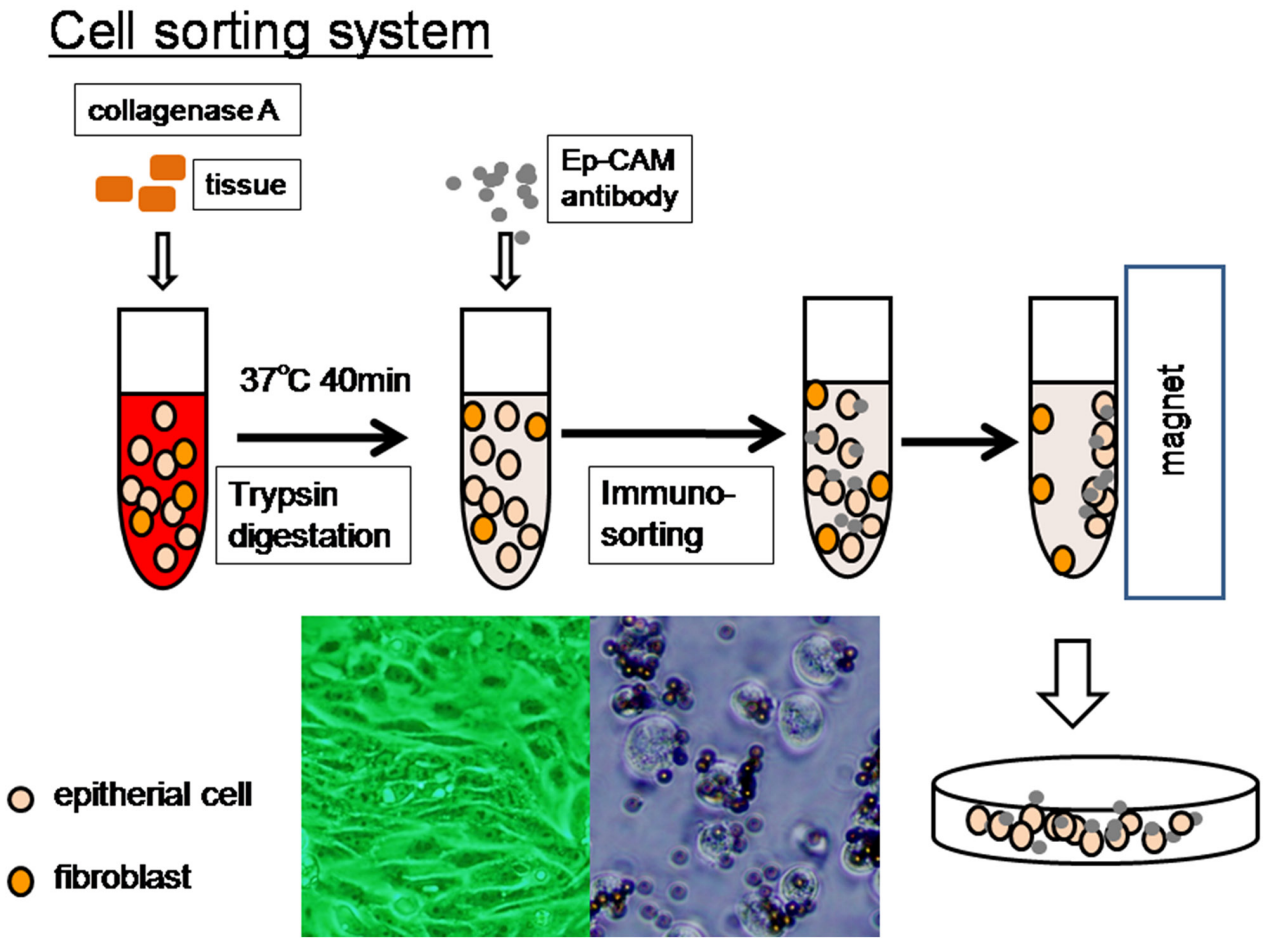
Overview of liquid microdissection technique Fresh tumor tissues were minced to 2-3-mm fragments and liquefied with collagenase A (10 mg/ml) with mild rolling at 37°C for 40 min. Tumor cells were isolated, after the large tissue fragments sunk to the bottom of the tube, using magnetic beads coated with an Ep-CAM antibody. Tumor cells were directly harvested for genomic DNA isolation or cultured for 3 days to confirm that these cells maintained their epithelial characteristics.

Tissue samples were obtained from the Department of Obstetrics and Gynecology at Shimane University School of Medicine between 2008 and 2013. The acquisition of tumor tissues was approved by the Shimane University Institutional Review Board. The diagnoses were confirmed by a surgical pathologist before the tumor samples were harvested for experiments. All patients had endometriotic lesions in the abdominal cavity or had episodes of endometriosis. There were three patients in stage 4. Metastatic sites were not present invital organs such as liver and lung. Patients exhibited only lymphnode metastasis at distant lesions such as the mediastinum lymphnode or carcinomatous pleural effusion. All patients were primarily treated with cytoreductive surgery and adjuvant platinum and taxane chemotherapy (carboplatin AUC5, paclitaxel 175 mg/m^2^ or docetaxel 70 mg/m^2^) or platinum and topoisomerase inhibitor chemotherapy (cisplatin 60 mg/m^2^, irinotecan 60 mg/m^2^). All patients received 6–12 courses of this regimen. When patients had disease recurrence, the second line chemotherapy regimen comprised PLD and CBDCA for platinum-sensitive patients and GEM, PLD, TPT alone, and bevacizumab for platinum-resistant patients, For sequencing analysis, tumor cells from 13 endometrioid carcinomas and nine clear cell carcinomas were isolated using the following protocol, which is illustrated in Figure 2.

The method used for cell sorting has been described in detail in our previous report [19]. Briefly, primary tumor cultures were derived from freshly isolated tumor samples by immune-sorting. Fresh tumor tissues were minced to 2-3-mm fragments and liquefied with collagenase A (10 mg/ml), with mild rolling at 37°C for 40 min. Small tumor cell clusters (< 10 cells) were gathered from the top part of centrifuge tubes after the large tissue fragments sank to the bottom of the tube. Tumor cells were isolated using magnetic beads coated with an Ep-CAM-specific antibody (Dynal, Oslo, Norway), anddirectly harvested for genomic DNA isolation. A small number of tumor cells were cultured for three days; we confirmed that these cells maintained the characteristics of epithelial cells.

### Mutational analysis in ovarian cancers by Sanger sequencing

Nucleotide sequencing was used to analyze the mutational status of *ARID1A, p53, POLE, PTEN, PIK3CA*, and *KRAS* in tumor cells isolated from ovarian carcinomas. In this study, we focused on analyzing exons that have been reported to harbor the majority of mutations for each of the genes. The primer sequences and the PCR protocol have been described in our previous reports [15, 19].

DNA was extracted and amplified by polymerase chain reaction with primers for exon 2 of *KRAS*, exons 1–9 of *PTEN*, exons 9 and 20 of *PIK3CA*, exons 1–9 of *p53*, exons 9–14 of *POLE*, and the whole exome sequence of *ARID1A.* Gene mutations were analyzed using primers described in Supplementary Table 1.

All mutations identified in the tumors were confirmed by independent PCR and Sanger sequencing in the specific tumors and their paired normal tissue to determine their somatic nature. Sequencing was performed using the ABI BigDye Terminator v3.1 Cycle Sequencing kit (Applied Biosystems, Thermo Fisher K. K Yokohama Japan). We also performed sequencing analysis to detect mutations in benign tissue such as blood from each patient with cancer. We determined whether the same mutations were present in both tumor and benign tissue to ascertain germ line mutations.

In the current study, we did not use NGS as the aim was to determine whether the frequency of mutations was altered based on the quality of DNA; therefore, it was not necessary to attempt to identify new hot spots and mutation sites.

### Statistical analysis of clinicopathological correlations

Statistical analyses were conducted using the Statistical Package for the Social Sciences for Windows software, Version 19.0 (IBM Corp., Armonk, NY, USA). All reported *P*-values were two-sided and *P*-values below 0.05 were considered to represent statistical significance.

Analysis of *POLE* and other gene mutations or alteration frequencies with clinicopathological parameters was performed using the chi-squared test.

Overall survival (OS) was defined as time from surgery to death from any cause. Patients were examined at the time of last follow-up. Progression-free survival (PFS) was defined as the time from surgery to the first recurrence or death from disease. For PFS analysis, patients were examined if they were alive with or without disease at the time of last follow-up. The data were plotted as Kaplan–Meier curves, and the statistical significance was determined by the log-rank test.

## SUPPLEMENTARY MATERIALS FIGURE AND TABLES







## Abbreviations

OCCC: ovarian clear cell carcinoma
OEC: ovarian endometrioid carcinoma
ERONs: endometriosis-related ovarian neoplasms
TCGA: The Cancer Genome Atlas
POLE: polymerase epsilon
ARID1A: AT-rich interactive domain 1A
EC: endometrial cancer
