# Supplementary material for: Affinity-purified DNA-based mutation profiles of endometriosis-related ovarian neoplasms in Japanese patients
Source: Oncotarget. 2018 Feb 22;9(19):14754–63. doi: 10.18632/oncotarget.24546 (PMC5871076; doi:10.18632/oncotarget.24546)
Supplement: Supplementary file 1 [file oncotarget-09-14754-s001.pdf]

Affinity-purified DNA-based mutation profiles of endometriosis-related ovarian neoplasms in Japanese patients

SUPPLEMENTARY MATERIALS

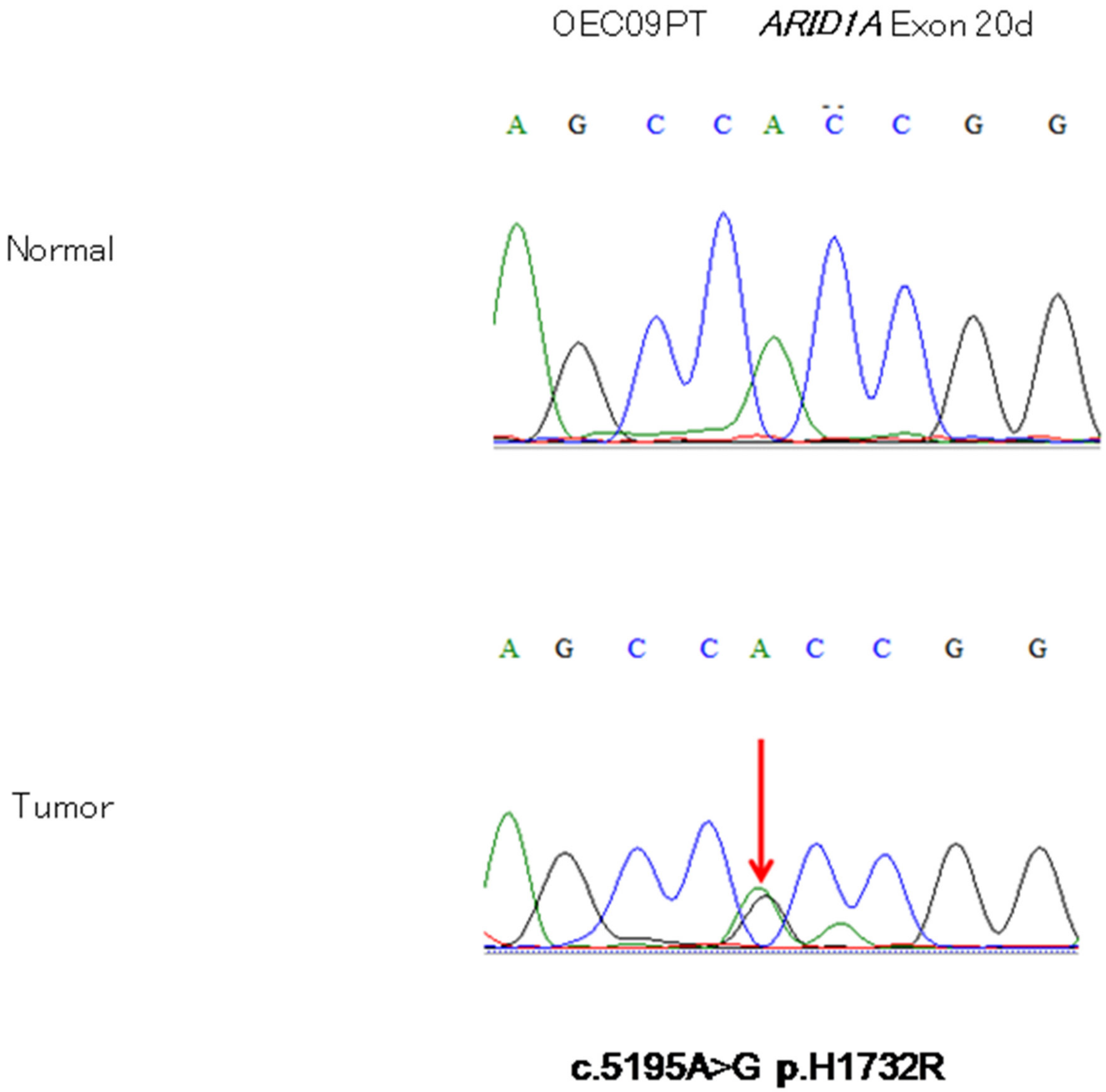

Supplementary Figure 1: Mutations were confirmed by Sanger sequencing using DNA from tumor and normal tissues from the same patients. There were no mutations in the same residues between tumor DNA and benign tissue DNA.

Supplementary Table 1: Sequencing PCR primer details.

See Supplementary File 1

Supplementary Table 2: Mutations identified in the discovery screen.

See Supplementary File 2
