# Supplementary material for: Affinity-purified DNA-based mutation profiles of endometriosis-related ovarian neoplasms in Japanese patients
Source: Oncotarget. 2018 Feb 22;9(19):14754–63. doi: 10.18632/oncotarget.24546 (PMC5871076; doi:10.18632/oncotarget.24546)
Supplement: Supplementary file 2 [file oncotarget-09-14754-s002.docx]

| **Supplementary Table 1: Sequencing PCR primer details** | | |
| --- | --- | --- |
|  |  |  |
| **Target (residues)** | **Primer name** | **Primer sequence** |
| *KRAS* | *KRAS*-Ex2-Fw | 5'-TTAACCTTATGTGTGACATGTTCTAA-3' |
| Exon2 | *KRAS*-Ex2-Rv | 5'-AGAATGGTCCTGCACCAGTAA-3' |
| *PTEN* | *PTEN*-Ex1-Fw | 5'-TTCCATCCTGCAGAAGAAGC-3' |
| Exon1 | *PTEN*-Ex1-Rv | 5'-CAGCCGCAGAAATGGATAC-3' |
| *PTEN* | *PTEN*-Ex2-Fw | 5'-ACTCCAGCTATAGTGGGGAAA-3' |
| Exon2 | *PTEN*-Ex2-Rv | 5'-TTTTCTGTGGCTTAGAAATCTTTT-3' |
| *PTEN* | *PTEN-*Ex3-Fw | 5'-TGATTACTACTCTAAACCCATAGAAGG-3' |
| Exon3 | *PTEN*-Ex3-Rv | 5'-TTGTTTTAGAAGATATTTGCAAGC-3' |
| *PTEN* | *PTEN*-Ex4-Fw | 5'-AAAGATTCAGGCAATGTTTGTT-3' |
| Exon4 | *PTEN*-Ex4-Rv | 5'-TCTCACTCGATAATCTGGATGAC-3' |
| *PTEN* | *PTEN*-Ex5-Fw | 5'-TCCAGTGTTTCTTTTAAATACCTGTT-3' |
| Exon5 | *PTEN*-Ex5-Rv | 5'-GATCCAGGAAGAGGAAAGGAA-3' |
| *PTEN* | *PTEN*-Ex6-Fw | 5'-ATATATGTTCTTAAATGGCTACGA-3' |
| Exon6 | *PTEN*-Ex6-Rv | 5'-ACATGGAAGGATGAGAATTTC-3' |
| *PTEN* | *PTEN*-Ex7-Fw | 5'-TCATTAAAATCGTTTTTGACAGTTT-3' |
| Exon7 | *PTEN*-Ex7-Rv | 5'-TCTGTCCTTATTTTGGATATTTCTC-3' |
| *PTEN* | *PTEN*-Ex8-Fw | 5'-TGTTTAACATAGGTGACAGATTTTCTT-3' |
| Exon8 | *PTEN*-Ex8-Rv | 5'-ACAAGTCAACAACCCCCACA-3' |
| *PTEN* | *PTEN*-Ex9-Fw | 5'-TGTTCATCTGCAAAATGGAATAA-3' |
| Exon9 | *PTEN*-Ex9-Rv | 5'-CACAATGTCCTATTGCCATTAAA-3' |
| *PIK3CA* | *PIK3CA*-Ex9-Fw | 5'-GGGAAAAATATGACAAAGAAAGC-3' |
| Exon9 | *PIK3CA*-Ex9-Rv | 5'-CTGAGATCAGCCAAATTCAGTT-3' |
| *PIK3CA* | *PIK3CA*-Ex20-Fw | 5'-CTCAATGATGCTTGGCTCTG-3' |
| Exon20 | *PIK3CA*-Ex20-Rv | 5'-TGGAATCCAGAGTGAGCTTTC-3' |
| *p53* | *p53*-Ex1-Fw | 5'- CCTTACTTGTCATGGCGACTG-3' |
| Exon1 | *p53*-Ex1-Rv | 5'-GAAAATACACGGAGCCGAGA-3' |
| *p53* | *p53*-Ex2/3-Fw | 5'- GGGTTGGAAGTGTCTCATGC-3' |
| Exon2/3 | *p53*-Ex2/3-Rv | 5'-AGCCCAACCCTTGTCCTTAC-3' |
| *p53* | *p53*-Ex4-Fw | 5'-CCTGGTCCTCTGACTGCTCT-3' |
| Exon4 | *p53*-Ex4-Rv | 5'-GCCAGGCATTGAAGTCTCAT-3' |
| *p53* | *p53*-Ex5-Fw | 5'-TCAGATAGCGATGGTGAGCA-3' |
| Exon5 | *p53*-Ex5-Rv | 5'-CTTAACCCCTCCTCCCAGAG-3' |
| *p53* | *p53*-Ex6-Fw | 5'-TCTGTCTCCTTCCTCTTCCTACA-3' |
| Exon6 | *p53*-Ex6-Rv | 5'-AACCAGCCCTGTCGTCTCT-3' |
| *p53* | *p53*-Ex7-Fw | 5'-ＣＴＴＧＧＧＣＣＴＧＴＧＴＴＡＴＣＴＣＣ-3' |
| Exon7 | *p53*-Ex7-Rv | 5'-ＧＧＧＴＣＡＧＡＧＧＣＡＡＧＣＡＧＡ-3' |
| *p53* | *p53*-Ex8-Fw | 5'-ＧＧＧＡＧＴＡＧＡＴＧＧＡＧＣＣＴＧＧＴ-3' |
| Exon8 | *p53*-Ex8-Rv | 5'-ＧＣＴＴＣＴＴＧＴＣＣＴＧＣＴＴＧＣＴＴ-3' |
| *p53* | *p53*-Ex9-Fw | 5'-ＧＧＡＧＡＣＣＡＡＧＧＧＴＧＣＡＧＴＴＡ-3' |
| Exon9 | *p53*-Ex9-Rv | 5'-ＣＣＣＣＡＡＴＴＧＣＡＧＧＴＡＡＡＡＣＡ-3' |
| *POLE* | *POLE*-Ex9-Fw | 5'-TGTTCAGGGAGGCCTAATGG-3' |
| Exon9 | *POLE*-Ex9-Rv | 5'-AACAAATAACTAACAGTGGGG-3' |
| *POLE* | *POLE*-Ex10-Fw | 5'-GCTGCAATTCTGATCTGACG-3' |
| Exon10 | *POLE*-Ex10-Rv | 5'-AACAAATAACTAACAGTGGGG-3' |
| *POLE* | *POLE*-Ex11-Fw | 5'-CTTCTGAACTTTGGGAGAGG-3' |
| Exon11 | *POLE*-Ex11-Rv | 5'-CACCTCCTAAGTCGACATGG-3' |
| *POLE* | *POLE*-Ex12-Fw | 5'-GCATTAGAGCCTGACCTGC-3' |
| Exon12 | *POLE*-Ex12-Rv | 5'-ACAGCACAGTCTGCAAGAGG-3' |
| *POLE* | *POLE*-Ex13-Fw | 5'-CGGGATGTGGCTTACGTGC-3' |
| Exon13 | *POLE*-Ex13-Rv | 5'-TTGCATCTGTCTGTGTGGTG-3' |
| *POLE* | *POLE*-Ex14-Fw | 5'-TCTGTGCTTCACACTTGACC-3' |
| Exon14 | *POLE*-Ex14-Rv | 5'-GACATCCACCTCCATTCAGC-3' |
| *ARID1A* | *ARID1A*-Ex1a-Fw | 5'-CCCGTTCGAGTTCTTCAGGT-3' |
| Exon1a | *ARID1A*-Ex1a-Rv | 5'-GCAGAAAGCGGAGAGTCACA-3' |
| *ARID1A* | *ARID1A*-Ex1b-Fw | 5'-GGGAAAGGAGCTGCAGGA-3' |
| Exon1b | *ARID1A*-Ex1b-Rv | 5'-ACCTCTCGGGGAGCTCAG-3' |
| *ARID1A* | *ARID1A*-Ex1c-Fw | 5'-CAGCAGAACTCTCACGACCA-3' |
| Exon1c | *ARID1A*-Ex1c-Rv | 5'-CCCACTCAGCTGTGTACCTG-3' |
| *ARID1A* | *ARID1A*-Ex1d-Fw | 5'-GAGAAGAGCCAGACAATGGC-3' |
| Exon1d | *ARID1A*-Ex1d-Rv | 5'-ACCCTCAACCAACTGCTCAC-3' |
| *ARID1A* | *ARID1A*-Ex 2a-Fw | 5'-TTGGAAGCCAAGGATACATT-3' |
| Exon2a | *ARID1A*-Ex 2a-Rv | 5'-AGGTTGGTCTCATTGCTCTTTC-3' |
| *ARID1A* | *ARID1A*-Ex 3a-Fw | 5'-ACCCTGGGCCTCCTAAGTATG-3' |
| Exon3a | *ARID1A*-Ex 3a-Rv | 5'-ATATCTTACCTGCGGTGGAGG-3' |
| *ARID1A* | *ARID1A*-Ex 3b-Fw | 5'-TGCACGTTAGAGAACCACTCTG-3' |
| Exon3b | *ARID1A*-Ex 3b-Rv | 5'-ACAACCAGCAAAGTCCTCACC-3' |
| *ARID1A* | *ARID1A*-Ex 4-Fw | 5'-CAGTCCCATAACCCTTTCACAG-3' |
| Exon4 | *ARID1A*-Ex 4-Rv | 5'-CTGGGCAGGGAGACAGAAC-3' |
| *ARID1A* | *ARID1A*-Ex 5-Fw | 5'-GAAACTATGCAGGCATGAGCC-3' |
| Exon5 | *ARID1A*-Ex 5-Rv | 5'-AAAGAACGTGTGTGATGTATTTGC-3' |
| *ARID1A* | *ARID1A*-Ex 6-Fw | 5'-TTGGCTGGATCTCTTTGTGTG-3' |
| Exon6 | *ARID1A*-Ex 6-RRv | 5'-TTCATGGTCAAACAGCTCTCC-3' |
| *ARID1A* | *ARID1A*-Ex 7-Fw | 5'-TCCCAGGATAAGGATGGAGAG-3' |
| Exon7 | *ARID1A*-Ex 7-Rv | 5'-GGACAGCCCTTCTCTCACAAG-3' |
| *ARID1A* | *ARID1A*-Ex 8-Fw | 5'-TTGAATGACATTGTTTGGTGTTC-3' |
| Exon8 | ARID1A-Ex 8-Rv | 5'-GGTCCAGAAGCATCTCAATAATC-3' |
| *ARID1A* | *ARID1A*-Ex 9-Fw | 5'-ATCATCTCTGGGCTGGCTG-3' |
| Exon9 | *ARID1A*-Ex 9-Rv | 5'-CACAGCACTATTTGGCTCCAG-3' |
| *ARID1A* | *ARID1A*-Ex 10-Fw | 5'-GGCTGGGATCTTGTCACTCTC-3' |
| Exon10 | *ARID1A*-Ex 10-Rv | 5'-GCCAACAATTCTGCAGGTAAG-3' |
| *ARID1A* | *ARID1A*-Ex 11-Fw | 5'-CAAGAGACTTCTGAGACCCTTAGC-3' |
| Exon11 | *ARID1A*-Ex 11-Rv | 5'-CATGGTACCACATGAAGCCAG-3' |
| *ARID1A* | *ARID1A*-Ex 12-Fw | 5'-ATCCTTGGCATATCCTGTTGG-3' |
| Exon12 | *ARID1A*-Ex 12-Rv | 5'-GAATACCTTACAGCCTGATGGG-3' |
| *ARID1A* | *ARID1A*-Ex 13-Fw | 5'-AACAAAGGACACGCAGGAGTC-3' |
| Exon13 | *ARID1A*-Ex 13-Rv | 5'-GGCCTTAGGAAGAACTTTCCC-3' |
| *ARID1A* | *ARID1A*-Ex 14-Fw | 5'-GGCTGAAGATAAGTGCATGGG-3' |
| Exon14 | *ARID1A*-Ex 14-Rv | 5'-CAAGAACCCTGAGCCATTCTC-3' |
| *ARID1A* | *ARID1A*-Ex 15-Fw | 5'-GAACTCTGAAGAGGGCCTGG-3' |
| Exon15 | *ARID1A*-Ex 15-Rv | 5'-AATTGGAGAGGCAGATTGAGC-3' |
| *ARID1A* | *ARID1A*-Ex 16-Fw | 5'-CAGAGTGAGGTAAGCATGACCC-3' |
| Exon16 | *ARID1A*-Ex 16-Rv | 5'-CCTTGGGTGGAGAACTGATTG-3' |
| *ARID1A* | *ARID1A*-Ex 17-Fw | 5'-GTGAGTAAAGCCTGGTCTCGG-3' |
| Exon17 | *ARID1A*-Ex 17-Rv | 5'-ATTGAGGACGTGGCTCTTCAG-3' |
| *ARID1A* | *ARID1A*-Ex 18a-Fw | 5'-GGAAGAAAGAGTGGTGGTTGC-3' |
| Exon18a | *ARID1A*-Ex 18a-Rv | 5'-CCAAACTGGAATGGAAATTGG-3' |
| *ARID1A* | *ARID1A*-Ex 18b-Fw | 5'-GGAGATGTACAGCGTGCCATA-3' |
| Exon18b | *ARID1A*-Ex 18b-Rv | 5'-TCGGTTCACGCCATGATAG-3' |
| *ARID1A* | *ARID1A*-Ex 18c-Fw | 5'-GCTATGTGCGAGGCAGGTACT-3' |
| Exon18c | *ARID1A*-Ex 18c-Rv | 5'-GCTCAGCAAGGCACCATGT-3' |
| *ARID1A* | *ARID1A*-Ex 18d-Fw | 5'-ATTGCATGGCAATGAAGGAG-3' |
| Exon18d | *ARID1A*-Ex 18d-Rv | 5'-CCTCCATCTAACTACCAGCCC-3' |
| *ARID1A* | *ARID1A*-Ex 19-Fw | 5'-TGGCTAAAGATGAGACATTCCC-3' |
| Exon19 | *ARID1A*-Ex 19-Rv | 5'-AGACAGAAACTGCCTTCCACC-3' |
| *ARID1A* | *ARID1A*-Ex 20a-Fw | 5'-GTCTTGCTCTCGAAGTGGGTC-3' |
| Exon20a | *ARID1A*-Ex 20a-Rv | 5'-GGAGAACCTTTGGGAAAGGAG-3' |
| *ARID1A* | *ARID1A*-Ex 20b-Fw | 5'-GGCTTCGAATGGTATTGGACA-3' |
| Exon20b | *ARID1A*-Ex 20b-Rv | 5'-CAGGCAAGGACAAGCCAG-3' |
| *ARID1A* | *ARID1A*-Ex 20c-Fw | 5'-GGCGAGTGTAACCAAGGTGTT-3' |
| Exon20c | *ARID1A*-Ex 20c-Rv | 5'-GCTAAGAGTTCAGAGGCCATCA-3' |
| *ARID1A* | *ARID1A*-Ex 20d-Fw | 5'-GCTAAGAGTTCAGAGGCCATCA-3' |
| Exon20d | *ARID1A*-Ex 20d-Rv | 5'-CCGCATCATGTCCACACTA-3' |
| *ARID1A* | *ARID1A*-Ex 20e-Fw | 5'-CCTTGGTTACACTCGCCAAC-3' |
| Exon20e | *ARID1A*-Ex 20e-Rv | 5'-CAGCCGTGATTCGTACAGAGTA-3' |
| *ARID1A* | *ARID1A*-Ex 20f-Fw | 5'-GAGGTGGAAGGAGGAGAGAGA-3' |
| Exon20f | *ARID1A*-Ex 20f-Rv | 5'-CTCAGTGACCGAAAGAACCC-3' |
