# Supplementary material for: Affinity-purified DNA-based mutation profiles of endometriosis-related ovarian neoplasms in Japanese patients
Source: Oncotarget. 2018 Feb 22;9(19):14754–63. doi: 10.18632/oncotarget.24546 (PMC5871076; doi:10.18632/oncotarget.24546)
Supplement: Supplementary file 3 [file oncotarget-09-14754-s003.docx]

| **Supplementary Table 2: Mutations identified in the discovery screen** | | | |  |
| --- | --- | --- | --- | --- |
| Samples | Gene | Nucleotide (cDNA) | Amino acid (protein) | Mutation type |
| OEC01PT | *ARID1A* | c.4450A>T | p. I1484F | Missense |
| OEC02PT | *ARID1A* | c.4119C>T | p P1373L | Missense |
|  | *ARID1A* | c.4125C>T | p P1375L | Missense |
| OEC03PT | *ARID1A* | c.5457A>T | p. E1819D | Missense |
|  | *ARID1A* | c.5612_5613insG | fs | fs |
| OEC04PT | *ARID1A* | c.728C>A | p. A243E | Missense |
|  | *ARID1A* | c.5209_5210 insG | fs | fs |
|  | *ARID1A* | c.5416G>A | p.E1806K | Missense |
| OEC6PT | *ARID1A* | c.908G>C | p.S303Y | Missense |
|  | *ARID1A* | c.960G>C | p.S320Y | Missense |
|  | *ARID1A* | c.4847_4848 ins A | fs | fs |
|  | *ARID1A* | c.5195A>G | p.H1732R | Missense |
|  | *ARID1A* | c.5632_5633 insG | fs | fs |
| OEC7PT | *ARID1A* | c.658A>G | p. S220G | Missense |
|  | *ARID1A* | c.899C>G | p. T300R | Missense |
|  | *ARID1A* | c.4453A>T | p. M1485L | Missense |
|  | *ARID1A* | c4125_4126 insA | fs | fs |
|  | *ARID1A* | c.5195A>G | p. H1732R |  |
|  | *ARID1A* | c.5480_5483delAGG | fs | fs |
|  | *ARID1A* | c.5485G>A | p. V1829M | Missense |
| OEC8PT | *ARID1A* | c.1914C>G | p. S638R | Missense |
|  | *ARID1A* | c.1918C>G | p. P640A | Missense |
|  | *ARID1A* | c.2020_2022 insTA | fs | fs |
|  | *ARID1A* | c.2873C>G | p. S958C | Missense |
| OCC9PT | *ARID1A* | c.2271C>A | p. P757H | Missense |
| OCC10PT | *ARID1A* | c.475_476 ins C | fs | fs |
|  | *ARID1A* | c.4453A>T | p. M1485L | Missense |
| OCC11PT | *ARID1A* | c.2023A>T | p. N675Y | Missense |
|  | *ARID1A* | c.2224A>G | p. N742D | Missense |
| OCC12PT | *ARID1A* | c.2240C>G | p. A747G | Missense |
|  | *ARID1A* | c.3298C>T | p. L1100F | Missense |
| OCC13PT | *ARID1A* | c.1445T>C | p. Q482R | Missense |
|  | *ARID1A* | c.5195A>G | p. H1732R | Missense |
| OCC14PT | *ARID1A* | c.730G>A | p. A244T | Missense |
|  | *ARID1A* | c.1712G>A | p. S571X | Nonsense |
| OCC15PT | *ARID1A* | c.4119C>T | p. P1373L | Missense |
|  | *ARID1A* | c.4124C>T | p. P1375L | Missense |
| OCC16PT | *ARID1A* | c.1788_1789insA | fs | fs |
|  | *ARID1A* | c.3323A>T | p. E1108V | Missense |
| OCC18PT | *ARID1A* | c.4157A>T | p. K1386M | Missense |
| OCC21PT | *ARID1A* | c.708_709 insA | fs | fs |
|  | *ARID1A* | c.2275A>G | p. M759V | Missense |
| OCC22PT | *ARID1A* | c.704_705 insC | fs | fs |
|  | *ARID1A* | c.731C>A | p.A244E | Missense |
|  | *ARID1A* | c.4453A>T | p. M1485L | Missense |
|  | *ARID1A* | c.5632_5633 insG | fs | fs |
| OEC03PT | *p53* | c.394A>G | p.L132E | Missense |
| OEC04PT | *p53* | c.743G>A | p.R248Q | Missense |
| OEC09PT | *p53* | c.215C>G | p.P72R | Missense |
| OEC011PT | *p53* | c.215C>G | p.P72R | Missense |
| OEC013PT | *p53* | c.215C>G | p.P72R | Missense |
| OEC019PT | *p53* | c.215C>G | p.P72R | Missense |
| OEC03PT | *PTEN* | c.746T>G | p.V249G | Missense |
| OEC06PT | *PTEN* | c.746T>G | p.V249G |  |
| OEC06PT | *PTEN* | c.1547T>A | p.V516E | Missense |
| OEC07PT | *PTEN* | c.637G>T | p.E213X | Missense |
| OEC08PT | *PTEN* | c.428_429insG | fs | fs |
| OEC09PT | *PTEN* | c.756T>G | p.D252E | Missense |
| OEC011PT | *PTEN* | c.427T>A | p.S143T | Missense |
| OEC012PT | *PTEN* | c.428_429insG | fs | fs |
| OEC015PT | *PTEN* | c.701G>A | p.R243Q | Missense |
| OEC015PT | *PTEN* | c.710A>G | p.K237R | Missense |
| OEC016PT | *PTEN* | c.628_629insC | fs | fs |
| OEC018PT | *PTEN* | c.424 delA | fs | fs |
| OEC019PT | *PTEN* | c.424 delA | fs | fs |
| OEC021PT | *PTEN* | c.628_629insC | fs | fs |
| OEC022PT | *PTEN* | c.630T>G | p.F210L | Missense |
| OEC01PT | *POLE* | c.1302T>C | p. Y434H | Missense |
| OEC03PT | *POLE* | c.1302T>C | p. Y434H | Missense |
| OEC04PT | *POLE* | c.1302T>C | p. Y434H | Missense |
| OEC08PT | *POLE* | c.1302T>C | p. Y434H | Missense |
| OEC010PT | *POLE* | c.1302T>C | p. Y434H | Missense |
| OEC011PT | *POLE* | c.1126G>T | p. A376S | Missense |
| OEC014PT | *POLE* | c.1126G>T | p. A376S | Missense |
| OEC016PT | *POLE* | c.1302T>C | p. Y434H | Missense |
| OEC018PT | *POLE* | c.1152G>T | p.Q384H | Missense |
| OEC018PT | *POLE* | c.1155G>T | p.Q385X | Missense |
| OEC018PT | *POLE* | c.1258_1259insG | fs | fs |
| OEC06PT | *PIK3CA* | c.3140A>T | p.H1047L | Missense |
| OEC07PT | *PIK3CA* | c.1633G>A | p.E545L | Missense |
| OEC016PT | *PIK3CA* | c.3140A>G | p.H1047R | Missense |
| OEC09PT | *KRAS* | c.35 G>T | p.G12V | Missense |
